# Supplementary material for: Functional characterization of SMARCA4 variants identified by targeted exome-sequencing of 131,668 cancer patients
Source: Nat Commun. 2020 Nov 3;11:5551. doi: 10.1038/s41467-020-19402-8 (PMC7609548; doi:10.1038/s41467-020-19402-8)
Supplement: Supplementary file 1 — Supplementary Information [file 41467_2020_19402_MOESM1_ESM.pdf]

Supplementary Information

Functional characterization of *SMARCA4* variants identified by targeted exome-sequencing of 131,668 cancer patients

Fernando et al.

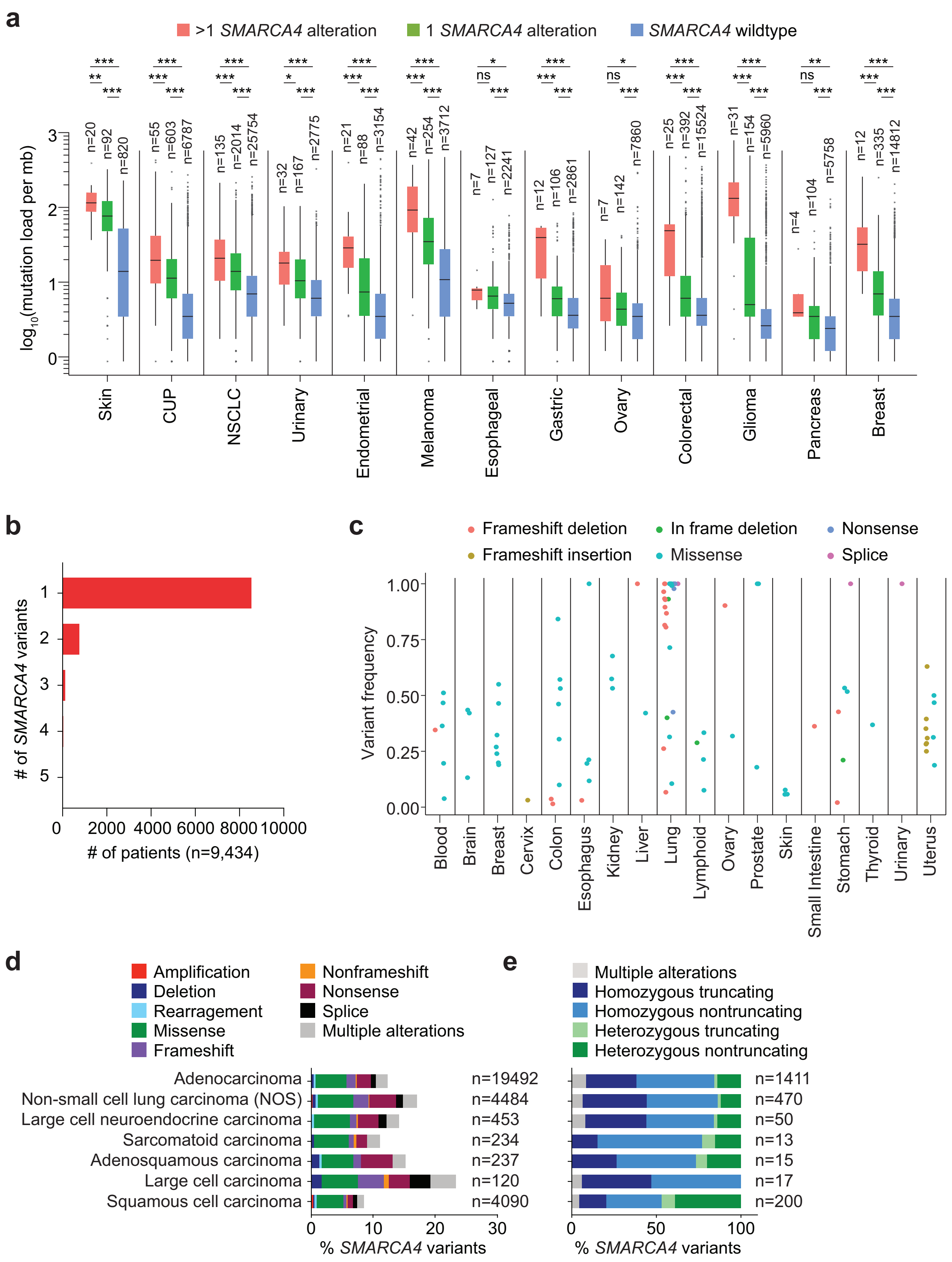

a
>1 SMARCA4 alteration
1 SMARCA4 alteration
SMARCA4 wildtype
log10(mutation load per mb)
Skin
CUP
NSCLC
Urinary
Endometrial
Melanoma
Esophageal
Gastric
Ovary
Colorectal
Glioma
Pancreas
Breast
b
# of SMARCA4 variants
# of patients (n=9,434)
c
Frameshift deletion
In frame deletion
Nonsense
Frameshift insertion
Missense
Splice
Variant frequency
Blood
Brain
Breast
Cervix
Colon
Esophagus
Kidney
Liver
Lung
Lymphoid
Ovary
Prostate
Skin
Small Intestine
Stomach
Thyroid
Urinary
Uterus
d
Amplification
Deletion
Rearrangement
Missense
Frameshift
Nonframeshift
Nonsense
Splice
Multiple alterations
Adenocarcinoma n=19492
Non-small cell lung carcinoma (NOS) n=4484
Large cell neuroendocrine carcinoma n=453
Sarcomatoid carcinoma n=234
Adenosquamous carcinoma n=237
Large cell carcinoma n=120
Squamous cell carcinoma n=4090
% SMARCA4 variants
e
Multiple alterations
Homozygous truncating
Homozygous nontruncating
Heterozygous truncating
Heterozygous nontruncating
n=1411
n=470
n=50
n=13
n=15
n=17
n=200
% SMARCA4 variants

Supplementary Figure 1. Tumor mutation burden and variant distribution of *SMARCA4* mutant cancers

a) Tumor mutation burden of patients with one or multiple alterations in *SMARCA4* relative to *SMARCA4* wildtype patients. The number of patients in each disease group with >1, 1 *SMARCA4* alteration or *SMARCA4* wildtype are indicated. First and third quartiles are represented by the lower and upper hinges with the median represented by the line within the box; whiskers extend to 1.5 x the interquartile range. Outliers are represented as individual points. Significance was tested with a two-tailed Wilcoxon rank-sum test (* $P<0.05$; ** $P<0.01$; *** $P<0.001$). Number of patients per group are indicated.
b) Number of *SMARCA4* variants per patient tumor.
c) Variation frequency of *SMARCA4* mutations in a panel of *SMARCA4* mutant cancer cell lines (n=98 cell lines).
d) Alteration frequency of *SMARCA4* in NSCLC subtypes. The number of patients in each NSCLC subtype assessed are indicated.
e) Zygosity of *SMARCA4* truncating and nontruncating variants in NSCLC subtype.

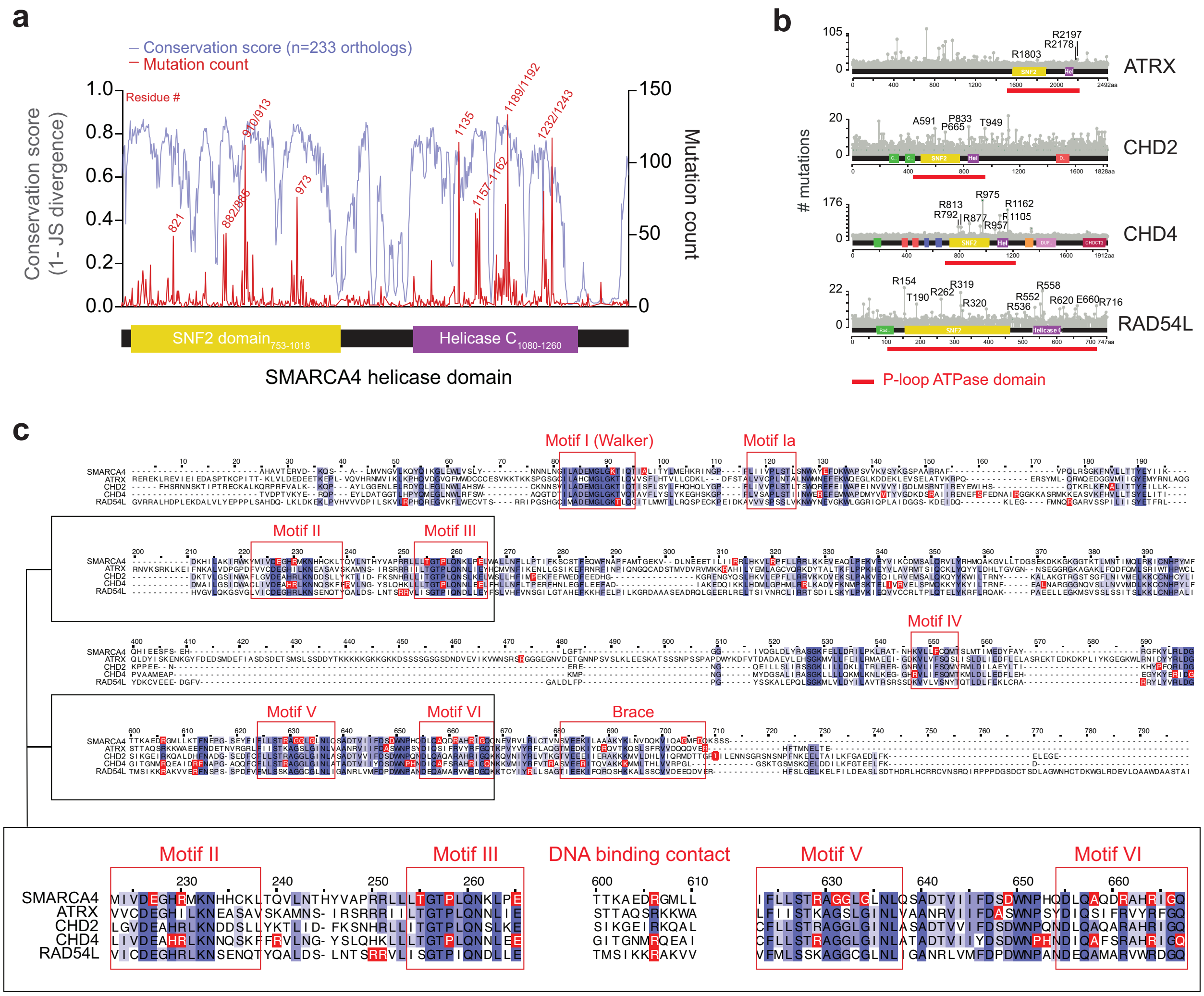

d

Residue	Count (n)	Domain
R1192	133	Helicase C-terminal
R1243	117	Helicase C-terminal
R1135	114	Helicase C-terminal
T910	112	Helicase ATP-binding
G1232	80	Helicase C-terminal
R973	76	Helicase hinge
R1189	71	Helicase C-terminal
G1162	68	Helicase C-terminal
R1157	65	Helicase C-terminal
G1159	61	Helicase C-terminal
R885	51	Helicase hinge
E882	50	Helicase ATP-binding
E821	49	Helicase ATP-binding
P913	45	Helicase ATP-binding
G1160	43	Helicase C-terminal
D1177	36	Helicase C-terminal
E920	36	Helicase ATP-binding
D1235	33	Helicase C-terminal
F1102	32	Helicase C-terminal
R966	32	Helicase ATP-binding
G1194	31	Helicase C-terminal
A791	29	Helicase ATP-binding
A1186	26	Helicase C-terminal
R979	21	Helicase hinge
K785R	-	ATPase dead, control

Supplementary Figure 2. Conservation and characterization of SMARCA4 mutations

a) Conservation and mutation count of SMARCA4 missense mutations. Sequence conservation is represented as 1-the Jensen-Shannon (JS) divergence using a 3-residue averaging window.

b) Lollipop plots of hotspot missense mutations of SNF2 family members profiled in the FoundationOne® panel (ATRX, CHD2, CHD4 and RAD54L) (n=131,668 patients).

c) ClustalW alignment of SMARCA4 and other SNF2 family members from (b). Residues in red signify where missense mutations occur. SNF2 members profiled in the FoundationOne® panel were used due to the cohort size (n=131,668), which allows for the identification of more missense mutations.

d) Table of frequently mutated residues of SMARCA4 protein. Mutations in red signify those chosen for functional validation. The ATPase dead K785R mutation was included as a control.

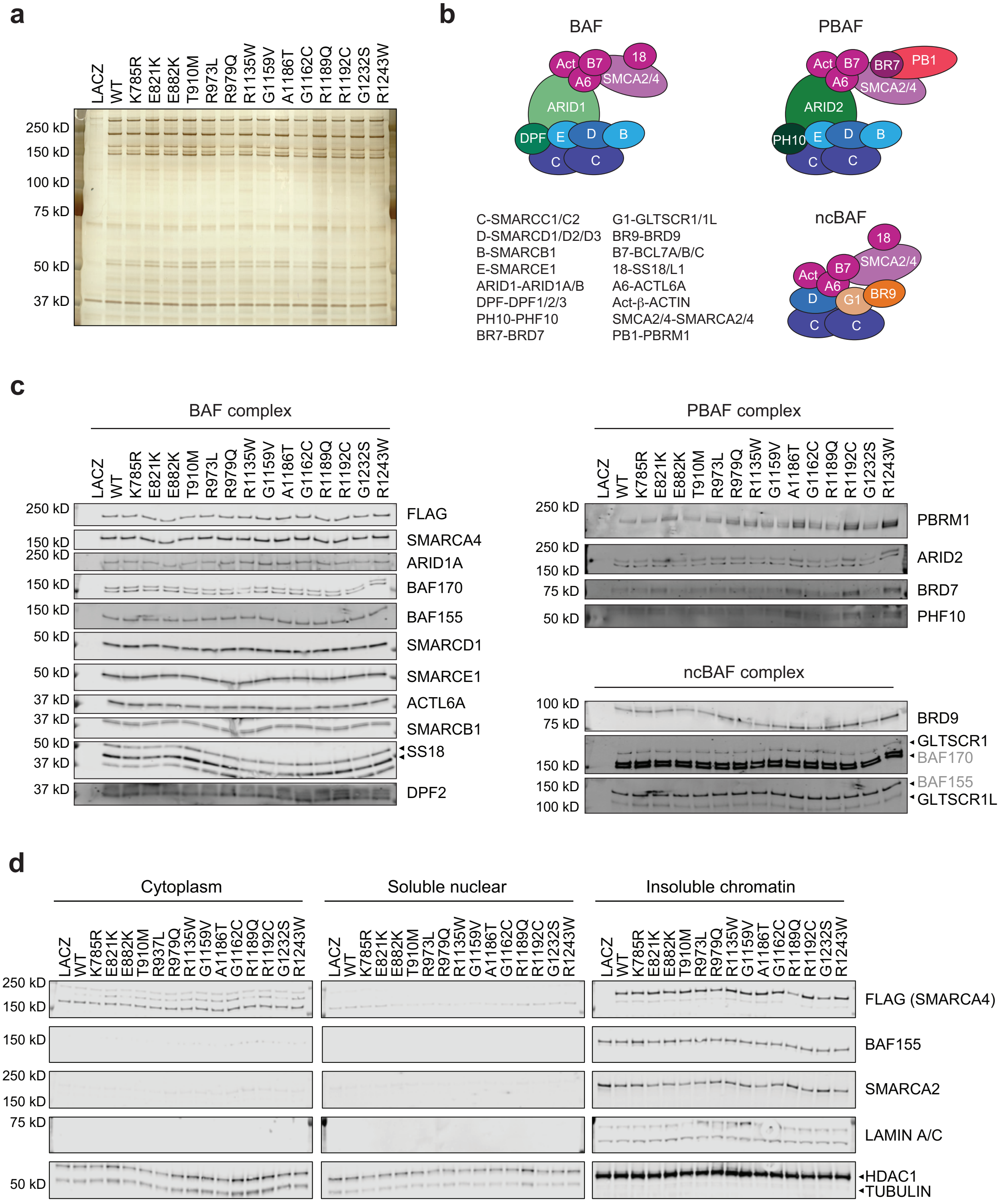
a
LACZ
WT
K785R
E821K
E882K
T910M
R973L
R979Q
R1135W
G1159V
A1186T
G1162C
R1189Q
R1192C
G1232S
R1243W
250 kD
150 kD
100 kD
75 kD
50 kD
37 kD
b
BAF
PBAF
ncBAF
Act
B7
18
A6
SMCA2/4
ARID1
DPF
E
D
B
C
BR7
PB1
ARID2
PH10
G1
BR9
C-SMARCC1/C2
D-SMARCD1/D2/D3
B-SMARCB1
E-SMARCE1
ARID1-ARID1A/B
DPF-DPF1/2/3
PH10-PHF10
BR7-BRD7
G1-GLTSCR1/1L
BR9-BRD9
B7-BCL7A/B/C
18-SS18/L1
A6-ACTL6A
Act-β-ACTIN
SMCA2/4-SMARCA2/4
PB1-PBRM1
c
BAF complex
PBAF complex
ncBAF complex
FLAG
SMARCA4
ARID1A
BAF170
BAF155
SMARCD1
SMARCE1
ACTL6A
SMARCB1
SS18
DPF2
PBRM1
ARID2
BRD7
PHF10
BRD9
GLTSCR1
BAF170
BAF155
GLTSCR1L
d
Cytoplasm
Soluble nuclear
Insoluble chromatin
R937L
FLAG (SMARCA4)
BAF155
SMARCA2
LAMIN A/C
HDAC1
TUBULIN

Supplementary Figure 3. SMARCA4 mutants do not affect BAF complex formation or localization

a) Silver staining of SMARCA4 FLAG immunoprecipitations from 293T cells transduced with SMARCA4 WT or mutants (representative of at least 2 replicates).
b) Composition of BAF, PBAF and ncBAF members.
c) Immunoblot of SMARCA4 FLAG immunoprecipitations for BAF, PBAF and ncBAF members in cells from (a) (representative of 3 replicates).
d) Immunoblot of cytoplasmic, nuclear and chromatin compartments of 293T transduced with SMARCA4 WT or mutants (representative of 2 replicates).

Source data are provided as a Source Data file.

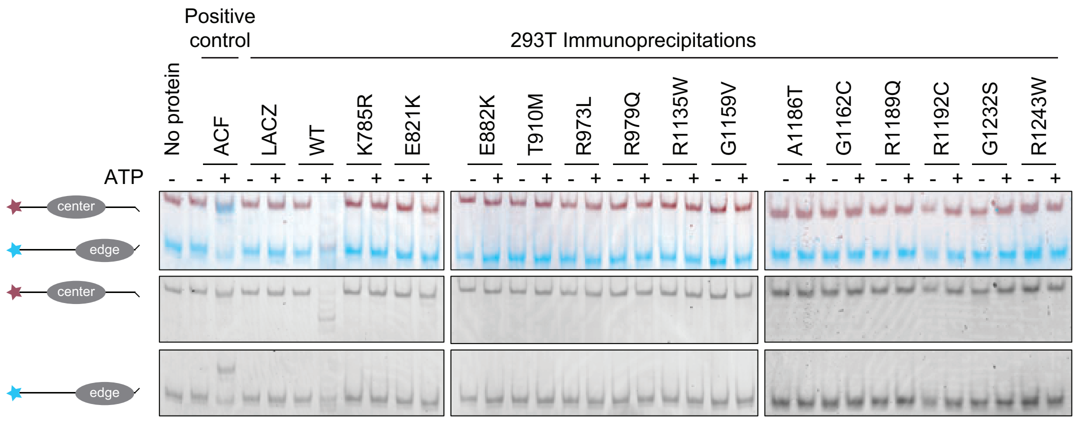

Supplementary Figure 4. SMARCA4 mutants cannot slide edge- or center-positioned nucleosomes

Gel shift assay of center- and edge-positioned nucleosomes with immunopurified SMARCA4 WT and mutants from 293T cells. Recombinant ACF is used for a positive control and only remodels edge-positioned nucleosomes. This experiment is representative of two replicates with the same result. Source data are provided as a Source Data file.

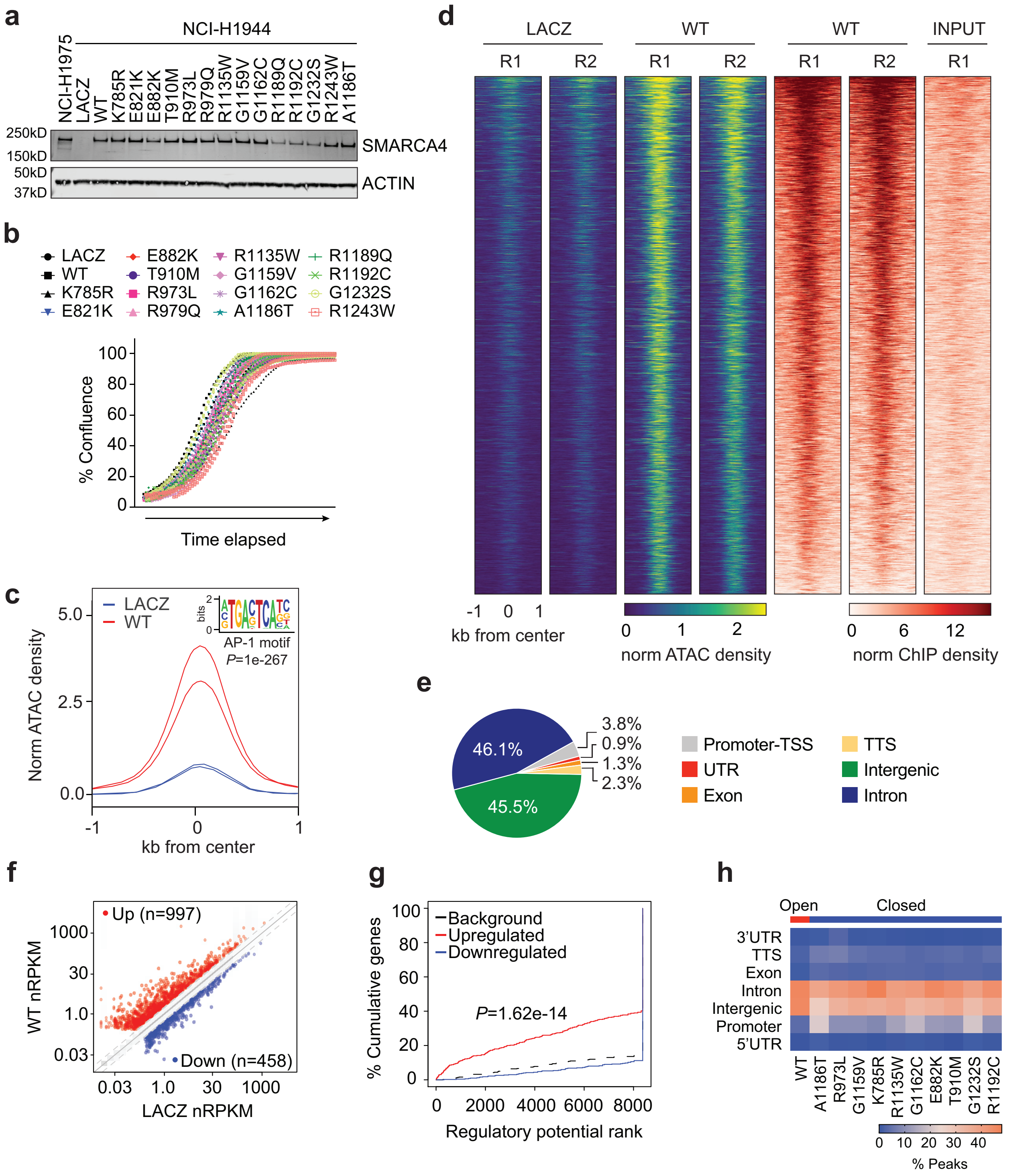

a
NCI-H1944
NCI-H1975
LACZ
WT
K785R
E821K
E882K
T910M
R973L
R979Q
R1135W
G1159V
G1162C
R1189Q
R1192C
G1232S
R1243W
A1186T
250kD
150kD
SMARCA4
50kD
37kD
ACTIN
b
LACZ
WT
K785R
E821K
E882K
T910M
R973L
R979Q
R1135W
G1159V
G1162C
A1186T
R1189Q
R1192C
G1232S
R1243W
% Confluence
100
80
60
40
20
0
Time elapsed
c
LACZ
WT
AP-1 motif
P=1e-267
Norm ATAC density
5.0
2.5
0.0
-1
0
1
kb from center
d
LACZ
WT
WT
INPUT
R1
R2
R1
R2
R1
R2
R1
-1 0 1
kb from center
0 1 2
norm ATAC density
0 6 12
norm ChIP density
e
46.1%
45.5%
3.8%
0.9%
1.3%
2.3%
Promoter-TSS
UTR
Exon
TTS
Intergenic
Intron
f
Up (n=997)
Down (n=458)
WT nRPKM
1000
30
1.0
0.03
0.03
1.0
30
1000
LACZ nRPKM
g
Background
Upregulated
Downregulated
P=1.62e-14
% Cumulative genes
100
80
60
40
20
0
0
2000
4000
6000
8000
Regulatory potential rank
h
Open
Closed
3'UTR
TTS
Exon
Intron
Intergenic
Promoter
5'UTR
WT
A1186T
R973L
G1159V
K785R
R1135W
G1162C
E882K
T910M
G1232S
R1192C
0 10 20 30 40
% Peaks

Supplementary Figure 5. SMARCA4 WT induces large increases in chromatin accessibility and gene expression

a) Immunoblot of NCI-H1944 cells transduced with SMARCA4 WT or mutants (representative of 3 replicates).

b) Growth rates of cells from (b) over the course of 10 days (n=2 technical replicates; representative of 3 independent experiments).

c) Normalized ATAC-seq read density of NCI-H1944 cells transduced with LACZ or SMARCA4 WT. Each line represents a replicate (n=2). AP-1 was the most significantly enriched motif identified by HOMER in regions opened by SMARCA4 WT using a hypergeometric test (one-tailed).

d) Heatmap of accessibility (ATAC-seq) and SMARCA4 occupancy by ChIP-seq at regions with increased accessibility in LACZ or WT cells from (a). Data are shown for each replicate (R) as normalized peak counts per million genomic DNA fragments (n=2). Rows are rank ordered by ATAC-seq peaks in WT.

e) Genomic distribution of chromatin accessible sites gained after SMARCA4 re-expression in cells from (a).

f) Scatter plot of gene expression changes in NCI-H1944 cells expressing LACZ or SMARCA4 WT. Significance was tested with a moderated t-statistic (two-sided) and *P* values were adjusted for multiple testing with the Benjamini–Hochberg procedure ($\log_2$ fold-change>1, adjusted *P*<0.05; n=3 per group). Gene expression was obtained in the form of normalized Reads Per Kilobase gene model per Million total reads (nRPKM).

g) BETA analysis graphs depicting the effect of differentially open ATAC sites in cells from (f). Genes were ranked from high to low according to the regulatory potential of the corresponding chromatin peak. Purple lines represent WT-downregulated genes while red lines represent WT-upregulated genes. A one-tailed Kolmogorov–Smirnov test was used to determine whether the up and downregulated groups differed significantly from the static group of transcriptionally unchanged genes (dashed lines).

h) Genomic distribution of ATAC sites opened after SMARCA4 WT (red bar) or closed after SMARCA4 mutant expression (blue bar) in cells from (a).

Source data are provided as a Source Data file.

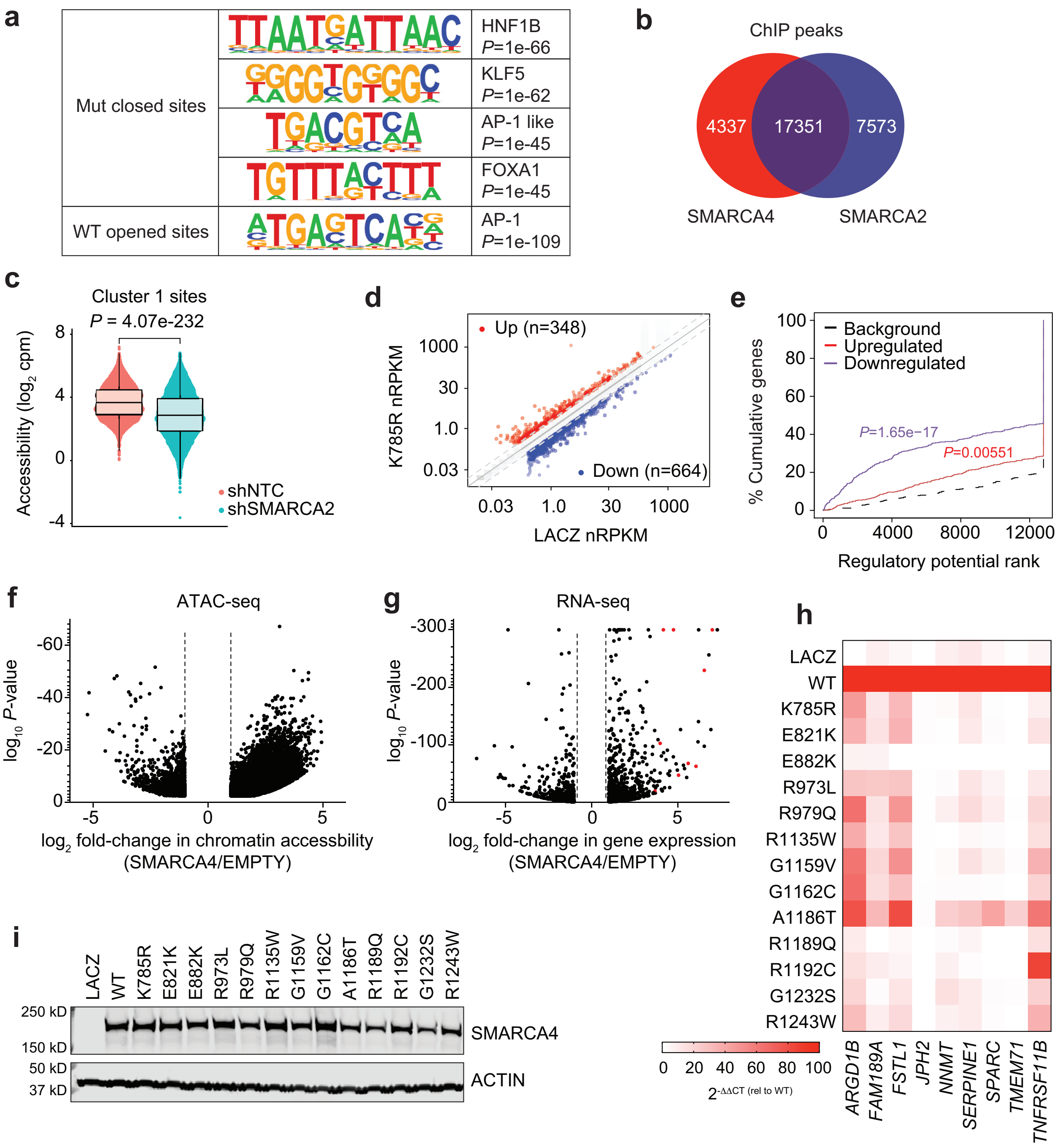

a
Mut closed sites
HNF1B P=1e-66
KLF5 P=1e-62
AP-1 like P=1e-45
FOXA1 P=1e-45
WT opened sites
AP-1 P=1e-109
b
ChIP peaks
4337
17351
7573
SMARCA4
SMARCA2
c
Cluster 1 sites
P = 4.07e-232
Accessibility (log2 cpm)
shNTC
shSMARCA2
d
Up (n=348)
Down (n=664)
K785R nRPKM
LACZ nRPKM
e
Background
Upregulated
Downregulated
P=1.65e−17
P=0.00551
% Cumulative genes
Regulatory potential rank
f
ATAC-seq
log10 P-value
log2 fold-change in chromatin accessbility (SMARCA4/EMPTY)
g
RNA-seq
log10 P-value
log2 fold-change in gene expression (SMARCA4/EMPTY)
h
LACZ
WT
K785R
E821K
E882K
R973L
R979Q
R1135W
G1159V
G1162C
A1186T
R1189Q
R1192C
G1232S
R1243W
ARGD1B
FAM189A
FSTL1
JPH2
NNMT
SERPINE1
SPARC
TMEM71
TNFRSF11B
2-ΔΔCT (rel to WT)
i
250 kD
150 kD
50 kD
37 kD
SMARCA4
ACTIN

Supplementary Figure 6. SMARCA4 mutants close a subset of SMARCA2-regulated sites and downregulate genes

a) Top motifs found enriched by HOMER in ATAC sites that closed after NCI-H1944 cells were transduced with SMARCA4 mutants or opened after transduced with SMARCA4 WT. A hypergeometric test (one-tailed) was used to determine *P* values.

b) Overlap of SMARCA2 and SMARCA4 ChIP-seq peaks. SMARCA2 ChIP was performed in NCI-H1944 cells transduced with LACZ. SMARCA4 ChIP was performed in NCI-H1944 cells transduced with SMARCA4 WT.

c) Violin plot of accessibility changes after *SMARCA2* knockdown of sites that closed after SMARCA4 mutant expression in NCI-H1944 cells (cluster 1 in Fig. 4e). First and third quartiles are represented by the lower and upper hinges with the median represented by the line within the box; whiskers extend to 1.5 x the interquartile range. Outliers are represented as individual points. Data represented as $\log_2$ normalized read counts per million (cpm) at n=3028 sites. Significance was tested with a two-tailed Wilcoxon rank-sum test.

d) Scatter plot of gene expression changes in NCI-H1944 cells transduced with LACZ or SMARCA4 K785R. Significance was tested with a moderated t-statistic (two-sided) and *P* values were adjusted for multiple testing with the Benjamini–Hochberg procedure ($\log_2$ fold-change>1, adjusted *P*<0.05; n=3 per group). Gene expression was obtained in the form of normalized Reads Per Kilobase gene model per Million total reads (nRPKM).

e) BETA analysis graphs depicting the effect of differentially closed ATAC sites in cells from (d) (n=2 per construct). Genes were ranked from high to low according to the regulatory potential of the corresponding chromatin peak. Purple lines represent K785R-downregulated genes, while red lines represent K785R-upregulated genes. A one-tailed Kolmogorov–Smirnov test was used to determine whether the up and downregulated groups differed significantly from the static group of transcriptionally unchanged genes (dashed lines).

f) ATAC-seq changes in NCI-H1299 cells transduced with empty vector or SMARCA4 WT. Significance was tested with a moderated t-statistic (two-sided) and P values were adjusted for multiple testing with the Benjamini–Hochberg procedure ($\log_2$ fold-change ≥1, adjusted *P*<0.05; n=3 per group).

g) Volcano plot of gene expression changes in cells from (f). Significance was tested with a moderated t-statistic (two-sided) and P values were adjusted for multiple testing with the Benjamini–Hochberg procedure ($\log_2$ fold-change>1, adjusted *P*<0.05; n=3 per group). Red dots indicate genes that increased expression, had increased accessibility changes and tested in (h).

h) Heatmap of qRT-PCR analysis of a subset of SMARCA4 WT-induced genes (red dots indicated in g) in NCI-H1299 transduced with SMARCA4 WT or mutants (mean of n=2 biologically independent samples).

i) Immunoblot of cells from (h) (representative of 2 replicates).

Source data are provided as a Source Data file.

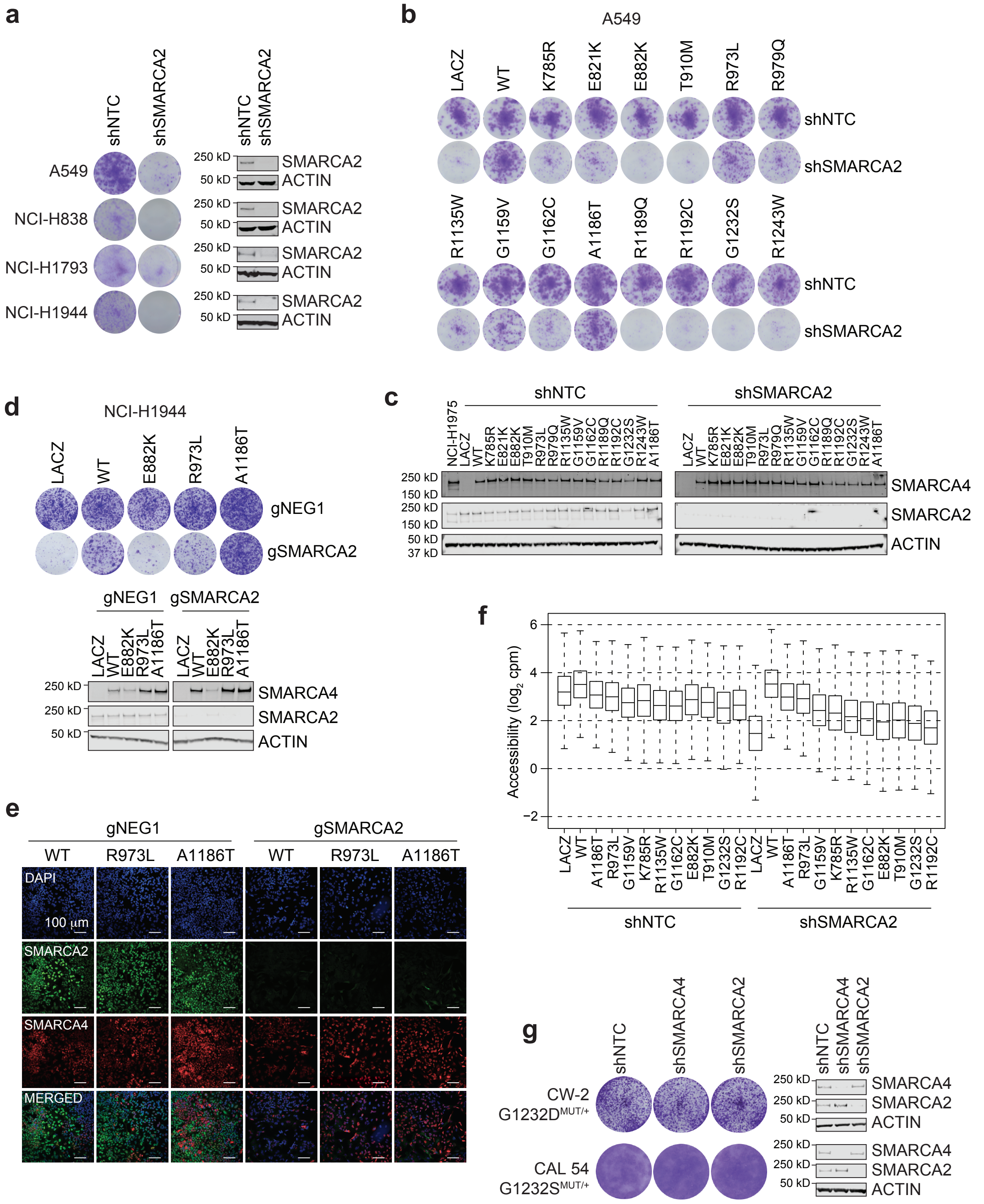
a
shNTC
shSMARCA2
A549
NCI-H838
NCI-H1793
NCI-H1944
250 kD
50 kD
SMARCA2
ACTIN
b
A549
LACZ
WT
K785R
E821K
E882K
T910M
R973L
R979Q
R1135W
G1159V
G1162C
A1186T
R1189Q
R1192C
G1232S
R1243W
shNTC
shSMARCA2
c
NCI-H1975
250 kD
150 kD
50 kD
37 kD
SMARCA4
SMARCA2
ACTIN
d
NCI-H1944
gNEG1
gSMARCA2
e
DAPI
100 µm
SMARCA2
SMARCA4
MERGED
f
Accessibility (log2 cpm)
6
4
2
0
−2
g
shSMARCA4
CW-2
G1232D^MUT/+
CAL 54
G1232S^MUT/+

Supplementary Figure 7. Cell viability and accessibility changes after *SMARCA2* knockdown in lung cancer cells

a) Long term clonogenic growth of lung cancer cell lines harboring homozygous *SMARCA4* truncating mutations after *SMARCA2* knockdown (representative of at least 3 replicates).

b) Long term clonogenic growth of A549 cells transduced with SMARCA4 WT or mutants after *SMARCA2* knockdown (representative of at least 3 replicates).

c) Immunoblot of cells from (b) (representative of at least 3 replicates).

d) Long term growth (top) and immunoblot (bottom) of NCI-H1944 cells transfected with negative control (gNEG1) or *SMARCA2*-targeting CRISPR/Cas9 ribonucleoprotein complexes after reconstitution with SMARCA4 WT or mutants (representative of 2 replicates).

e) Immunofluorescence of cells from (d) that grew out after CRISPR/Cas9-mediated knockout of *SMARCA2* (representative of 2 replicates).

f) Box plots of accessibility at sites lost after *SMARCA2* knockdown in NCI-H1944 cells transduced with SMARCA4 WT or mutants after *SMARCA2* knockdown. First and third quartiles are represented by the lower and upper hinges with the median represented by the line within the box; whiskers extend to 1.5 x the interquartile range. Data represented as $\log_2$ normalized read counts per million (cpm) at n=7939 sites.

g) Long term growth of CW-2 and CAL 54, which all harbor heterozygous *SMARCA4* missense mutations, after knockdown of *SMARCA2* or *SMARCA4* (left). Immunoblot confirming SMARCA2/SMARCA4 protein depletion (right). Representative of two replicates.

Source data are provided as a Source Data file.
