# Supplementary material for: Functional characterization of SMARCA4 variants identified by targeted exome-sequencing of 131,668 cancer patients
Source: Nat Commun. 2020 Nov 3;11:5551. doi: 10.1038/s41467-020-19402-8 (PMC7609548; doi:10.1038/s41467-020-19402-8)
Supplement: Supplementary file 3 — Description of Additional Supplementary Files [file 41467_2020_19402_MOESM3_ESM.pdf]

Description of Additional Supplementary Files

File Name: Supplementary Data 1
Description**: SMARCA4 variants in the FoundationCORE® database.** SMARCA4 short variants, copy number alterations and rearrangements detected from 9,434 patients with at least 1 SMARCA4 alteration (n=10,562 SMARCA4 variants).

File Name: Supplementary Data 2
Description**: Summary of total samples and SMARCA4 variants separated by cancer type.** The disease ontology of the total number of samples profiled, those with at least one SMARCA4 variant and those with SMARCA4 variants that could be assessed for zygosity.

File Name: Supplementary Data 3
Description**: SMARCA4 variants in human tumor-derived cell lines.** SMARCA4 variants identified by exome-sequencing in human tumor-derived cell lines.

File Name: Supplementary Data 4
Description: **qRT-PCR and ChIP reagents used for all experiments.** Taqman expression assays and sequences for qChIP primers.
