# Supplementary material for: Functional characterization of SMARCA4 variants identified by targeted exome-sequencing of 131,668 cancer patients
Source: Nat Commun. 2020 Nov 3;11:5551. doi: 10.1038/s41467-020-19402-8 (PMC7609548; doi:10.1038/s41467-020-19402-8)
Supplement: Supplementary file 8 — Reporting Summary [file 41467_2020_19402_MOESM8_ESM.pdf]

nature research

Corresponding author(s): Robert Yauch

Last updated by author(s): October 2, 2020

Reporting Summary

Nature Research wishes to improve the reproducibility of the work that we publish. This form provides structure for consistency and transparency in reporting. For further information on Nature Research policies, see Authors & Referees and the Editorial Policy Checklist .

Statistics

For all statistical analyses, confirm that the following items are present in the figure legend, table legend, main text, or Methods section.

n/a	Confirmed	
☐	☒	The exact sample size (n) for each experimental group/condition, given as a discrete number and unit of measurement
☒	☐	A statement on whether measurements were taken from distinct samples or whether the same sample was measured repeatedly
☐	☒	The statistical test(s) used AND whether they are one- or two-sided<br>*Only common tests should be described solely by name; describe more complex techniques in the Methods section.*
☒	☐	A description of all covariates tested
☐	☒	A description of any assumptions or corrections, such as tests of normality and adjustment for multiple comparisons
☐	☒	A full description of the statistical parameters including central tendency (e.g. means) or other basic estimates (e.g. regression coefficient) AND variation (e.g. standard deviation) or associated estimates of uncertainty (e.g. confidence intervals)
☐	☒	For null hypothesis testing, the test statistic (e.g. F, t, r) with confidence intervals, effect sizes, degrees of freedom and P value noted<br>*Give P values as exact values whenever suitable.*
☒	☐	For Bayesian analysis, information on the choice of priors and Markov chain Monte Carlo settings
☐	☒	For hierarchical and complex designs, identification of the appropriate level for tests and full reporting of outcomes
☐	☒	Estimates of effect sizes (e.g. Cohen's d, Pearson's r), indicating how they were calculated

Our web collection on statistics for biologists contains articles on many of the points above.

Software and code

Policy information about availability of computer code

Data collection	Immunoblot: Image Studio version 3.1<br>FRET: Tecan i-control software common version 3.7.3.0<br>qPCR/qChIP: Applied Biosystems FLEX<br>Cell confluence: Incucyte<br>Gel shift: Typhoon<br>IF: Opera Phenix<br>NGS: Illumina NextSeq 500 and HiSeq4000
Data analysis	R version 3.5.1<br>GSNAP version 2013-10-10; http://research-pub.gene.com/gmap/<br>MACS2 version 2.1.0<br>Limma R package version 3.38.3<br>HTSeqGenie R package version 4.12.0<br>HOMER version 4.7<br>BETA version 1.0.7<br>Prism 8 version 8.3.0<br>Pymol version 2.0<br>Clustal Omega version 1.2.2

For manuscripts utilizing custom algorithms or software that are central to the research but not yet described in published literature, software must be made available to editors/reviewers. We strongly encourage code deposition in a community repository (e.g. GitHub). See the Nature Research guidelines for submitting code & software for further information.

Data

Policy information about availability of data

All manuscripts must include a data availability statement. This statement should provide the following information, where applicable:

- Accession codes, unique identifiers, or web links for publicly available datasets
- A list of figures that have associated raw data
- A description of any restrictions on data availability

All ATAC/ChIP/RNA-seq data that support the findings of this study have been deposited in the Gene Expression Omnibus (GEO) with accession code "GSE144844 [https://www.ncbi.nlm.nih.gov/geo/query/acc.cgi?acc=GSE144844]". Full variant information for ~18,000 samples have been deposited in the Genomics Data Commons (GDC) with study accession "phs001179 [https://gdc.cancer.gov/about-gdc/contributed-genomic-data-cancer-research/foundation-medicine/foundation-medicine]". In an effort to minimize any risk of re-identification of individuals with respect to the Health Insurance Portability and Accountability Act, additional detailed data will not be provided. However, all SMARCA4 variants described in this study are found in Supplementary Data 1. Source data for Figures 4a, 4f, 4g and Supplementary Figures 5b, 6h are provided with this paper. The remaining data are available within the Article, Supplementary Information or available from the authors upon request.

Field-specific reporting

Please select the one below that is the best fit for your research. If you are not sure, read the appropriate sections before making your selection.

[x] Life sciences [ ] Behavioural & social sciences [ ] Ecological, evolutionary & environmental sciences

For a reference copy of the document with all sections, see nature.com/documents/nr-reporting-summary-flat.pdf

Life sciences study design

All studies must disclose on these points even when the disclosure is negative.

Sample size	No sample size calculations were performed. ATAC- and ChIP-seq studies were performed in biological duplicate. RNA-seq studies were performed in biological triplicate. Other experiments were performed with two or more biological replicates as reported in the Statistics and Reproducibility section and figure legends. Samples sizes were chosen empirically in line with previous publications that performed similar techniques (Pan et al., Nature Genetics, 2019; Michel et al., Nature Cell Biology, 2018).
Data exclusions	No data exclusions were performed.
Replication	FRET and gel shift assays were replicated twice, with each orthogonal method confirming the same result. SMARCA4 immunoprecipitations followed by silver stains and immunoblots were replicated at least twice. qPCR of gene induction after SMARCA4 WT- and mutant-reconstitution was replicated at least twice and confirmed in 2 different cell lines. qChIP experiments were replicated at least 3 times with similar results. Incucyte confluence measurements and colony forming assays in SMARCA4 WT- and mutant-reconstituted cell lines with and without SMARCA2 knockdown were replicated at least 3 times. Colony forming assays and immunofluorescence in SMARCA4 WT- and mutant-reconstituted cells after CRISPR knockout of SMARCA2 were replicated twice. ATAC- and ChIP-seq were performed in duplicate; RNA-seq were performed in triplicate. ChIP- and RNA-seq was validated in a panel of genes using qChIP and qPCR experiments. ATAC-seq and RNA-seq after SMARCA4 WT reconstitution was performed in 2 different cell lines, showing similar results.
Randomization	Randomization was not relevant to this study as all experiments were performed with molecular/cell biology techniques in cell lines where the experimenter designs and performs the experimental conditions so randomization between conditions is not possible.
Blinding	Blinding was not relevant to this study as experiments were performed with molecular/cell biology techniques in cell lines where the experimenter designs and performs the experimental conditions so blinding is not possible.

Reporting for specific materials, systems and methods

We require information from authors about some types of materials, experimental systems and methods used in many studies. Here, indicate whether each material, system or method listed is relevant to your study. If you are not sure if a list item applies to your research, read the appropriate section before selecting a response.

Materials & experimental systems

n/a	Involved in the study	
[ ]	[x]	Antibodies
[ ]	[x]	Eukaryotic cell lines
[x]	[ ]	Palaeontology
[x]	[ ]	Animals and other organisms
[ ]	[x]	Human research participants
[x]	[ ]	Clinical data

Methods

n/a	Involved in the study	
[ ]	[x]	ChIP-seq
[x]	[ ]	Flow cytometry
[x]	[ ]	MRI-based neuroimaging

Antibodies

Antibodies used	Immunoblot: Primary (1:1000 antibody dilutions): SMARCA4/BRG1 (Abcam ab110641); SMARCA2/BRM (Cell Signaling Technologies, 11966); SMARCC1/BAF155 (Cell Signaling Technologies, 11956S); SMARCC2/BAF170 (Bethyl, A301-039A); BAF47/SNF5 (Cell Signaling Technologies, 91735); SMARCD1/BAF60A (Bethyl, A301-595A); SMARCE1/BAF57 (Bethyl, A300-810A); ACTL6A/BAF53A (Bethyl, A301-391A); ARID1A/BAF250A (Cell Signaling Technologies, 12354); ARID1B/BAF250B (Bethyl, A301-047A); SS18 (Cell Signaling Technologies, 21792), DPF2 (Abcam, ab134942); PBRM1/BAF180 (Millipore, ABE370); ARID2 (Santa Cruz, E-3); ARID2 (Bethyl, A302-230A); BRD7 (Cell Signaling Technologies, 15125); PHF10 (Abcam, ab154637); BRD9 (Abcam, ab137245); GLTSCR1 (Invitrogen, PA5-63267); GLTSCR1L (Novus, NBP1-86359); ACTIN (Cell Signaling Technologies, 3700), TUBULIN (Cell Signaling Technologies, 2148), HDAC1 (Cell Signaling Technologies, 34589), LAMIN A/C (Cell Signaling Technologies, 4777), FLAG (Sigma, M2). Secondary (1:1000 antibody dilution): goat anti-mouse IgG conjugated to IRDye 680RD (Licor, 926-68070); goat anti-rabbit IgG conjugated to IRDye 800CW (Licor, 926-65010).<br><br>Immunofluorescence: Primary antibodies: SMARCA2 (1:2000, Cell Signaling Technologies, 11966); SMARCA4 (1:500, Santa Cruz, G-7). Secondary antibodies (1:1000): goat anti-rabbit IgG F(ab')2 fragment conjugated with Alexa Fluor 488 (Cell Signaling Technologies, 4412) and goat anti-mouse IgG F(ab')2 fragment conjugated with Alexa Fluor 647 (Cell Signaling Technologies, 4410).<br><br>qChIP: SMARCA4 antibody (4uL/30ug chromatin, Abcam, ab110641).<br><br>ChIP-seq: SMARCA2 (5uL/30ug chromatin, Abcam, ab15597); SMARCA4 antibody (10uL/30ug chromatin, Abcam, ab110641).
Validation	The primary antibodies for ACTIN (cat no. 3700), TUBULIN (cat. no 2148), HDAC1 (cat no. 34589), LAMIN A/C (cat no. 4777) have been validated for the detection of human proteins by immunoblot, and validation data are available on the Cell Signaling Technology website. The primary antibody for FLAG (M2) has been validated for the immunoprecipitation and detection of FLAG-tagged proteins by immunoblot, and validation data are available on the Sigma website. The SMARCA4 (G-7) and SMARCA2 (cat no. 11966) primary antibodies have been validated for use in immunofluorescence in human cells, and validation data are available on the Santa Cruz and Cell Signaling Technology websites, respectively. The SMARCA4 (ab110641) and SMARCA2 (ab15597) antibodies have been validated for use in ChIP of human cells, and validation data are available on the Abcam website. Primary antibodies for other BAF subunits were chosen based on previous publications that also validated their specificity and application (Pan et al., Nature Genetics, 2019; Michel et al., Nature Cell Biology, 2018).

Eukaryotic cell lines

Policy information about cell lines

Cell line source(s)	A549, NCI-H838, NCI-H1299, NCI-H1435, NCI-H1793, NCI-H1944 and NCI-H1975 cells were obtained from ATCC. CW-2 cells were obtained from the Riken Institute. HCC1897 cells were obtained from University of Texas Southwestern Medical Center. CAL 54 and CAL-12T cells were obtained from DSMZ. Lenti-X 293T cells were obtained from Takara Bio.
Authentication	A549, NCI-H838, NCI-H1299, NCI-H1435, NCI-H1793, NCI-H1944, NCI-H1975, CW-2, HCC1897, CAL 54 and CAL-12T cell lines were authenticated using SNP genotyping and STR profiling by the Genentech internal cell line repository, gCell. Lenti-X 293T cells were not authenticated after being purchased from Takara Bio.
Mycoplasma contamination	All cell lines tested negative for mycoplasma.
Commonly misidentified lines<br>(See ICLAC register)	No commonly misidentified cell lines were used in this study.

Human research participants

Policy information about studies involving human research participants

Population characteristics	The samples represent an adult (>-18) 'all comers' pan-solid tumor cohort of patients profiled through Dec 2017 in the FoundationCORE database. Patients were 56% female, 44% male with a median age of 62 (inter-quartile range of 53-70). There is limited demographic data in the FoundationCORE database.
Recruitment	The samples used in this study were not specifically selected and represent an 'all comers' patient population to Foundation Medicine genomic profiling (pan-solid tumors and pan-center). The pathologic diagnosis of each case was confirmed by review of hematoxylin and eosin stained slides, and all samples that advanced to nucleic acid extraction contained a minimum of 20% tumor cells.
Ethics oversight	Approval was obtained from the Western Institutional Review Board (Protocol No. 20152817). Patients consented for the use of their data for analysis but not for raw data release.

Note that full information on the approval of the study protocol must also be provided in the manuscript.

ChIP-seq

Data deposition

☒ Confirm that both raw and final processed data have been deposited in a public database such as GEO.

☒ Confirm that you have deposited or provided access to graph files (e.g. BED files) for the called peaks.

Data access links<br>*May remain private before publication.*	To review GEO accession GSE144844:<br>Go to https://www.ncbi.nlm.nih.gov/geo/query/acc.cgi?acc=GSE144844<br>GSE147201 is the ChIP-seq SubSeries of the GSE144844 SuperSeries (which contains also ATAC-seq and RNA-seq).
Files in database submission	fastqs, bigWigs, peak files
Genome browser session<br>(e.g. UCSC)	No longer applicable

Methodology

Replicates	2 replicates per ChIP sample
Sequencing depth	Sample name Total reads High quality mapped reads Uniquely mapped<br>H1944-LACZ-NoDOX_SMARCA2_R1 44006980 42043070 34055648<br>H1944-LACZ-NoDOX_SMARCA2_R2 41274196 39557369 31730014<br>H1944-LACZ-NoDOX_SMARCA4_R1 38055184 36324563 26988090<br>H1944-LACZ-NoDOX_SMARCA4_R2 34702219 32889633 24252324<br>H1944-SMARCA4-NoDOX_SMARCA4_R1 44317965 42354735 33809081<br>H1944-SMARCA4-NoDOX_SMARCA4_R2 44129357 42237556 33921593<br>H1944-SMARCA4-DOX_SMARCA4_R1 32712293 31260566 25489385<br>H1944-SMARCA4-DOX_SMARCA4_R2 36183765 34711339 28707115<br>H1944-LACZ-NoDOX_Input 44865086 42961388 36444735<br>H1944-SMARCA4-NoDOX_Input 32617062 31202484 26475195<br>H1944-SMARCA4-DOX_Input 33255753 31744677 27043051<br><br>All reads were 75 bp single-end.
Antibodies	SMARCA2 (5uL/30ug chromatin, Abcam, ab15597 lot GR3180556-1); SMARCA4 antibody (10uL/30ug chromatin, Abcam, ab110641, lot GR3208604-7).
Peak calling parameters	Sequencing reads were aligned to the human reference genome (NCBI Build 38) using GSNAP37 version '2013-10-10', allowing a maximum of two mismatches per read sequence (parameters: '-M 2 -n 10 -B 2 -i 1 --pairmax-dna=1000 --terminal-threshold=1000 --gmap-mode=none --clip-overlap'). Mapped reads then were assessed for peaks relative to the input controls using Macs2 (v.2.1.0) callpeak function.<br><br>Macs commands:<br>macs2 callpeak -t H1944-LACZ-NoDOX_SMARCA2_R1.bam -c H1944-LACZ-NoDOX_Input.bam -f BAM -g 2.7e9 -n H1944-LACZ-NoDOX_SMARCA2_R1 --outdir macs_output<br>macs2 callpeak -t H1944-LACZ-NoDOX_SMARCA2_R2.bam -c H1944-LACZ-NoDOX_Input.bam -f BAM -g 2.7e9 -n H1944-LACZ-NoDOX_SMARCA2_R2 --outdir macs_output<br>macs2 callpeak -t H1944-LACZ-NoDOX_SMARCA4_R1.bam -c H1944-LACZ-NoDOX_Input.bam -f BAM -g 2.7e9 -n H1944-LACZ-NoDOX_SMARCA4_R1 --outdir macs_output<br>macs2 callpeak -t H1944-LACZ-NoDOX_SMARCA4_R2.bam -c H1944-LACZ-NoDOX_Input.bam -f BAM -g 2.7e9 -n H1944-LACZ-NoDOX_SMARCA4_R2 --outdir macs_output<br>macs2 callpeak -t H1944-SMARCA4-NoDOX_SMARCA4_R1.bam -c H1944-SMARCA4-NoDOX_Input.bam -f BAM -g 2.7e9 -n H1944-SMARCA4-NoDOX_SMARCA4_R1 --outdir macs_output<br>macs2 callpeak -t H1944-SMARCA4-NoDOX_SMARCA4_R2.bam -c H1944-SMARCA4-NoDOX_Input.bam -f BAM -g 2.7e9 -n H1944-SMARCA4-NoDOX_SMARCA4_R2 --outdir macs_output<br>macs2 callpeak -t H1944-SMARCA4-DOX_SMARCA4_R1.bam -c H1944-SMARCA4-DOX_Input.bam -f BAM -g 2.7e9 -n H1944-SMARCA4-DOX_SMARCA4_R1 --outdir macs_output<br>macs2 callpeak -t H1944-SMARCA4-DOX_SMARCA4_R2.bam -c H1944-SMARCA4-DOX_Input.bam -f BAM -g 2.7e9 -n H1944-SMARCA4-DOX_SMARCA4_R2 --outdir macs_output
Data quality	We followed Encode guidelines for data quality assessment. The target nonredundancy fraction (NRF) for all samples except H1944-LACZ_SMARCA4_R1 and R2 were >= 0.8 for 10 million reads. All samples except H1944-LACZ_SMARCA4_R1 and R2 had a fraction of reads in peaks (FRiP) greater than 1%. H1944-LACZ_SMARCA4 samples were SMARCA4 ChIP done in H1944 cells transduced with LACZ (and therefore do not express SMARCA4 WT). Hence ChIP'd DNA is minimal in these samples

leading to slightly lower NRFs (0.74) and FRiPs (0.4/0.8%) and lower number of peaks.

Samples Number of peaks (FDR<5%; fold-enrichment =>5)
H1944-LACZ-NoDOX_SMARCA2_R1 37397
H1944-LACZ-NoDOX_SMARCA2_R2 49014
H1944-LACZ-NoDOX_SMARCA4_R1 132
H1944-LACZ-NoDOX_SMARCA4_R2 3415
H1944-SMARCA4-NoDOX_SMARCA4_R1 35488
H1944-SMARCA4-NoDOX_SMARCA4_R2 58422
H1944-SMARCA4-DOX_SMARCA4_R1 36157
H1944-SMARCA4-DOX_SMARCA4_R2 42329

Software

Sequencing reads were aligned to the human reference genome (NCBI Build 38) using GSNAP37 version '2013-10-10', allowing a maximum of two mismatches per read sequence (parameters: '-M 2 -n 10 -B 2 -i 1 --pairmax-dna=1000 --terminal-threshold=1000 --gmap-mode=none --clip-overlap'). Mapped reads then were assessed for peaks relative to the input controls using Macs2 (v.2.1.0) callpeak function.
